# Supplementary material for: Pricing and coordination in a dual-channel supply chain with a socially responsible manufacturer
Source: PLoS One. 2020 Jul 29;15(7):e0236099. doi: 10.1371/journal.pone.0236099 (PMC7390389; doi:10.1371/journal.pone.0236099)
Supplement: S1 Data — (DOCX) [file pone.0236099.s001.docx]

data set

In this paper, we use game theory to study coordination contract design in a dual-channel supply chain with a socially responsible manufacturer. Thus, the readers can replicate the results of our study through the proofs in the appendix section.

We also use numerical example in this paper. The original data of parameters are: $\alpha=0.2$, $\lambda=0.2$, $\theta=0.7$, $c_{r}=0.3$, $c_{d}=0.2$. Substituting these data to equations we obtained in the paper, the readers can redraw the figures in this paper.
