# Supplementary material for: Pricing and coordination in a dual-channel supply chain with a socially responsible manufacturer
Source: PLoS One. 2020 Jul 29;15(7):e0236099. doi: 10.1371/journal.pone.0236099 (PMC7390389; doi:10.1371/journal.pone.0236099)
Supplement: S1 Appendix — (DOCX) [file pone.0236099.s002.docx]

**Appendix**

**Proof of theorem 1**

From (4), we have Hessian matrix of $V^{C}$,

$H=\left(\begin{matrix} \frac{\partial^{2}V^{C}}{\partial p_{r}^{2}},\frac{\partial^{2}V^{C}}{\partial p_{d}\partial p_{r}} \\ \frac{\partial^{2}V^{C}}{\partial p_{r}\partial p_{d}},\frac{\partial^{2}V^{C}}{\partial p_{d}^{2}} \end{matrix}\right)\to\left(\begin{matrix} -\frac{2-\alpha}{1-\theta},\text{ }\frac{2-\alpha}{1-\theta} \\ \frac{2-\alpha}{1-\theta},\text{ }-\frac{2-\alpha}{\theta\left(1-\theta\right)} \end{matrix}\right)$ (A1)

From (A1), we have $\left(-\frac{2-\alpha}{ 1-\theta}\right)\left(-\frac{2-\alpha}{\theta\left(1-\theta\right)}\right)-\left(\frac{2-\alpha}{ 1-\theta}\right)^{2}=\frac{\left(\alpha-2\right)^{2}}{\theta\left(1-\theta\right)}>0$ and $-\frac{2-\alpha}{1-\theta}<0$, which indicates Hessian matrix of $V^{C}$ is negative definite. Then solving (4), we have $p_{r}^{C*}$ and $p_{d}^{C*}$. Substituting these optimal decisions, we can obtain equilibrium results under the centralized scenario.

**Proof of theorem 2**

Under D scenario, we firstly solve $p_{r}$ due to backward induction. And then we can easily verify that $\pi_{r}^{D}$ is a quadratic function of $p_{r}$ and $\partial^{2}\pi_{r}^{D}/\partial p_{r}^{2}<0$, which indicates that there exists a unique solution. Let $\partial\pi_{r}^{D}/\partial p_{r}=0$, we have $p_{r}^{D*}=\left(1-\theta+p_{d}+w+ c_{r}\right)/2$.

Then we solve $p_{d}$ and $w$ without constraints. Substituting $p_{r}^{D*}$ into (6), we have Hessian matrix of $V_{m}^{D}$,

$H=\left(\begin{matrix} \frac{\partial^{2}V_{m}^{D}}{\partial w^{2}},\frac{\partial^{2}V_{m}^{D}}{\partial p_{d}\partial w} \\ \frac{\partial^{2}V_{m}^{D}}{\partial w\partial p_{d}},\frac{\partial^{2}V_{m}^{D}}{\partial p_{d}^{2}} \end{matrix}\right)\to\left(\begin{matrix} -\frac{8 \theta-2 \alpha\theta}{8\theta\left(1-\theta\right)},\text{ } \frac{8 \theta-2 \alpha\theta}{8\theta\left(1-\theta\right)} \\ \frac{8 \theta-2 \alpha\theta}{8\theta\left(1-\theta\right)},\text{ }\frac{4 \alpha-3 \alpha\theta+4 \theta-8}{4\theta\left(1-\theta\right)} \end{matrix}\right)$ (A2)

From (A2), we have $\left(-\frac{8 \theta-2 \alpha\theta}{8\theta\left(1-\theta\right)}\right)\cdot\frac{4 \alpha-3 \alpha\theta+4 \theta-8}{4\theta\left(1-\theta\right)}-\left(\frac{8 \theta-2 \alpha\theta}{8\theta\left(1-\theta\right)}\right)^{2}=\frac{\left(2-\alpha\right)\left(4-\alpha\right)}{4\theta\left(1-\theta\right)}>0$ and $-\frac{8 \theta-2 \alpha\theta}{8\theta\left(1-\theta\right)}<0$, which indicates that Hessian matrix of $V_{m}^{D}$ is negative definite. Then solving (5), we have $w^{D*}$ and $p_{d}^{D*}$. Substituting these optimal decisions into $p_{r}^{D*}$, we obtain $p_{r}^{D*}$. Due to $p_{d}>w$ and $\theta p_{r}>p_{d}$, then $C_{1}<c_{d}<C_{2}$ and $c_{r}>C_{3}$ should be satisfied. Then substituting these optimal decisions, we can get equilibrium results under the decentralized scenario.

**Proof of proposition 2**

Comparing equilibrium results between the centralized and decentralized scenarios, we have $p_{d}^{C*}=p_{d}^{D*}$, $p_{r}^{D*}-p_{r}^{C*}=\frac{2\left(1-\theta+c_{d}-c_{r}\right)}{\left(2-\alpha\right)\left(4-\alpha\right)}>0$, and $\pi_{SC}^{C*}-\pi_{SC}^{D*}=\frac{2 \left(1-\theta+c_{d}-c_{r}\right)^{2}\left(\alpha^{2}-4 \alpha+2\right)}{\left(1-\theta\right)\left(2-\alpha\right)^{2}\left(4-\alpha\right)^{2}}$. It is easy to find out $\text{sign}\left\{\pi_{SC}^{C*}-\pi_{SC}^{D*}\right\}=\text{sign}\left\{\alpha^{2}-4 \alpha+2\right\}$. Then we can derive that $\pi_{SC}^{C*}>\pi_{SC}^{D*}$ when $0\leq\alpha<2-\sqrt{2}$; $\pi_{SC}^{C*}<\pi_{SC}^{D*}$ otherwise. The proof of $SW^{C*}>SW^{D*}$ is similar to that mentioned before.

**Proof of theorem 3**

Due to (8), we obtain $p_{r}^{L*}=\frac{\lambda\theta-\lambda p_{d}-\lambda-\theta+w+c_{r}+p_{d}+1}{2\left(1-\lambda\right)}$.

Let $\Omega$ ($\Omega\geq0$) denote profit of the retailer after paying the franchise fee $F^{L*}$ to the manufacturer. Then $F^{L*}\text{=}\left(\left(1-\lambda\right)p_{r}-w-c_{h}\right)q_{r}-\Omega$. Substituting $p_{r}^{L*}$ and $F^{L*}$ into (8), we have

$\max_{p_{d},w} V_{m}^{L}=wq_{r}+\left(p_{d}-c_{d}\right)q_{d}+\lambda p_{r}q_{r}+\alpha CS+\left(\left(\left(1-\lambda\right)p_{r}-w-c_{h}\right)q_{r}-\Omega\right)$ (A3)

Solving optimization problem in (A3), we have $w^{L*}$ and $p_{d}^{L*}$. Then we have $p_{r}^{L*}$, $p_{r}^{L*}=p_{r}^{C*}$, and $p_{d}^{L*}=p_{d}^{C*}$. Substituting these optimal decisions, we can get supply chain members’ equilibrium profits after coordination under low CSR scenario.

*Proof of proposition 3.*

Under low CSR scenario, conditions that the manufacturer will offer revenue sharing contract with franchise fee are $\pi_{m}^{L*}\geq\pi_{m}^{D*}$ and $V_{m}^{L*}\geq V_{m}^{D*}$. Solving these conditions, we have $F^{L*}\geq f_{1}$. Condition that the retailer will accept revenue sharing contract with franchise fee is $\pi_{r}^{L*}\geq\pi_{r}^{D*}$. Solving these conditions, we have $F^{L*}\leq f_{2}$. It is easy to verify that $f_{1}>0$, $f_{2}>0$, and $f_{2}-f_{1}>0$. Then we have $f_{1}\leq F^{L*}\leq f_{2}$.
