# Supplementary material for: Pricing and coordination in a dual-channel supply chain with a socially responsible manufacturer
Source: PLoS One. 2020 Jul 29;15(7):e0236099. doi: 10.1371/journal.pone.0236099 (PMC7390389; doi:10.1371/journal.pone.0236099)
Supplement: Attachment — Submitted filename: Comment.pdf [file pone.0236099.s003.pdf]

The paper titled ‘Coordination contract design in a dual-channel supply chain where the manufacturer has corporate social responsibility?’ seems interesting to me and can be reported through PLOS ONE. However, I suggest that the authors introduce the following major comments to enrich the quality of the manuscript.

Comment 1: The title of the paper seems not okay to me. Modify it.

Comment 2: Please make sure that a competent editor checks the English. For example, for example, in Line 155 and 229, page 6 and Line 229 respectively. Use of the first person ("I", "we", etc.) and third person ("he", "she" etc.) must be avoided.

Comment 3: The author has used very long sentences due to which it creates a readability problem. Try to make maximum of 11 words.

Comment 3: In mathematical expression, small brackets have been used only. In present version, I think this might confuse the readers.

Comment 4: The major defect of the proposed model is that the numerical illustration and sensitivity analysis has not been demonstrated. Also, this study is a lack of managerial implications.

Comment 5: I would like to request the author to emphasis on the contributions on practically.

Comment 6: Line 168, page 7, consumer utility function has been taken. Please cite this equation from which the journal is adopted.

Comment 7: The conclusion section is not explained effectively manner. Rewrite it.

Comment 8: Literature review is not adequate.

The authors can cite the following interesting papers.

Cachon, G.P., Lariviere, M.A., 2005. Supply chain coordination with revenue-sharing contracts: strengths and limitations. Manage. Sci. 51, 30–44.

Ranjan, Amit, and J. K. Jha. "Pricing and coordination strategies of a dual-channel supply chain considering green quality and sales effort." *Journal of cleaner production* 218 (2019): 409-424.

Based on the aforementioned points, my opinion is in the favour of major revision of this article.
