# Supplementary material for: Pricing and coordination in a dual-channel supply chain with a socially responsible manufacturer
Source: PLoS One. 2020 Jul 29;15(7):e0236099. doi: 10.1371/journal.pone.0236099 (PMC7390389; doi:10.1371/journal.pone.0236099)
Supplement: Attachment — Submitted filename: 20200613response to the comments.docx [file pone.0236099.s004.docx]

**Authors’ Response to the Comments**

**Manuscript No.:** PONE-D-19-35719

**Title:** Coordination contract design in a dual-channel supply chain where the manufacturer has corporate social responsibility

We are grateful to the editors and the three anonymous reviewers for their constructive and thorough comments on our paper. We have seriously considered and addressed all the comments in the revised manuscript. Our point-to-point responses are as following. The original comments are cited in italic. To facilitate review, the major changes are highlighted in yellow in the revised manuscript. Please review these revisions.

**Reviewer 1:**

*In this paper, in a dual-channel supply chain composed of a socially responsible manufacturer and a retailer, two different contracts are designed to achieve supply chain coordination based on the manufacturer's CSR coefficient, which have certain practical significance. However, the following problems still exist:*

**Reply:** Thanks for your thorough comments and helpful suggestions. Following your suggestion, the issues have been addressed.

*1. There are at least two written errors in the manuscript. First, “qh” should be “qr” in equation 5 on page 8. Second, in lines 286 and 287 on page 12, "profit of the DCSC is lower while social welfare is higher than C scenario" should be "profit of the DCSC is lower while social welfare is higher than D scenario ".*

**Reply:** Thanks for your comment and we are sorry for our careless. We have corrected these mistakes in the revised manuscript. Besides, we also have checked the whole paper to avoid such mistakes.

*2. In the first two parts, the authors propose two coordination contracts in a DCSC in order to maximize social welfare, but the purpose is not clear when the contracts are proposed. The authors need to further clarify the reasons or the purposes of the coordination contracts design. Ideas of the authors should be clearer.*

**Reply:** Thanks for your suggestion. After reading the original manuscript carefully, we agree with you that the purpose of the two coordination contracts is not clear. Therefore, in the revised manuscript, we have added why the socially responsible manufacturer offers a coordination contract to the retailer and why the coordination contract is designed as two parts in section 5 (p.12). The modification is also given in the following.

Under C scenario, the whole supply chain obtains the optimal social welfare. However, under D scenario, the whole supply chain suffers social welfare loss due to individual rationality. As the leader of the supply chain, the socially responsible manufacturer has incentive to design a contract to coordinate the supply chain. Furthermore, with the influence of socially responsibility, the purpose of the manufacturer to design contract are twofold: (1) to achieve the optimal social welfare under C scenario; (2) to make sure profits of supply chain members are no less than those under D scenario. Therefore, how to design coordination contract to achieve the above two purposes is the main problem facing by the socially responsible manufacturer.

*3. As an important variable in this paper, the authors should briefly explain what social welfare means, in order to facilitate the readers' understanding.*

**Reply:** Thanks for your suggestion. We have added the explanation on social welfare in the revised manuscript (p.8). The modification is also given in the following.

Social welfare is defined as the sum of the firm's profit brought by selling products and consumer surplus [6, 26]. Thus, in our model, the total social welfare includes the supply chain's profit and consumer surplus.

*4. The analysis of propositions is not deep enough, especially proposition 2. The authors need to further combine the professional knowledge to analyze the causes of the results in order to enhance the persuasiveness of the results.*

**Reply:** Thanks for your suggestion. In the revised manuscript, we have revised analysis of propositions (p.11). The modification of proposition 2 is also given in the following.

Proposition 2 implies that selling price in online store under D scenario equals that under C scenario while retail price under D scenario is higher than retail price under C scenario. Proposition 2 also indicates that social welfare under D scenario is always lower than C scenario. The reason is as follows. Under C scenario, the social responsibility supply chain is an integrated entity, which makes decisions based on total utility maximization instead of profit maximization. Thus the social responsibility supply chain cuts the retail price to obtain optimal social welfare. Under D scenario, the socially responsible manufacturer makes decisions based on total utility maximization while the retailer is based on profit maximization. Due to the inconsistency of decision goals and double marginalization under D scenario, the DCSC suffers social welfare loss.

It is common sense that profit obtained under C scenario is always higher than that under D scenario in traditional supply chain. However, in the DCSC with a socially responsible manufacturer, when $\alpha<2-\sqrt{2}$, profit of the supply chain profit under C scenario is higher than that under D scenario; when $2-\sqrt{2}<\alpha<1$, profit of the supply chain profit under C scenario is lower than that under D scenario. The reason behind this phenomenon is as follows. With the increase of CSR coefficient of the manufacturer, profits of the supply chain decrease under both D and C scenarios. However, the descent speed of profit under C scenario is faster than that under D scenario.

To explain the effect of the manufacturer's CSR on the supply chain profits and social welfare under the two scenarios, we draw fig 2 and fig 3 based on $\theta=0.7$, $c_{r}=0.3$, and $c_{d}=0.2$.

Thank you again for your constructive comments and valuable suggestions which improve our paper.

**Reviewer 2:**

*This study considers coordination of a dual-channel SC under the manufacturer’s CSR effort. A wholesale price contract with franchise fee and government subsidy is applied to coordinate the SC.*

**Reply:** Thanks for your thorough comments and helpful suggestions. Following your suggestion, the issues have been addressed.

*1. What is the author’s meaning from “too much CSR” in the abstract? How much is “too much”?*

**Reply:** Thanks for your suggestion and we are sorry for the inappropriate expression. Actually, what we want to express is that when CSR coefficient is higher than $2-\sqrt{2}$. Thus, in the revised manuscript, we have clearly pointed out its meaning in the abstract (p.2).

*2. Some of findings are quit questionable and are not consistent with the literature at all. For example the authors’ model results that: “when the socially responsible manufacturer has high CSR coefficient, the DCSC obtains less revenue under the centralized scenario”. More profit for a centralized supply chain is a rule in the literature of supply chain coordination. Also this is a mathematical fact that the centralized system creates more profit than the decentralized one. I think something is wrong in the developed model which results such an odd outcome.*

**Reply:** Thanks for your suggestion. As you said, “the centralized system creates more profit than the decentralized one” in the normal cases. However, the manufacturer bears corporate social responsibility in this paper, i.e. the socially responsible manufacturer makes decisions based on social welfare maximization instead of profit maximization in the traditional case. Thus, we have the interesting conclusion, which is “social welfare under C scenario is always higher than that under D scenario; when CSR coefficient is low, profit under C scenario is higher than that under D scenario; when CSR coefficient is high, profit under C scenario is lower than that under D scenario”. This conclusion is also one of the main contribution of this paper.

To avoid misunderstanding, we have revised the abstract in the revised manuscript (p.2). The modification is also given in the following.

With the impact of CSR, social welfare under centralized scenario is always higher than that under decentralized scenario. However, profit of the whole supply chain between the two scenarios has different relationship. More specifically, when CSR coefficient is relatively lower than a certain threshold, profit under centralized scenario is higher than that under decentralized scenario. When CSR coefficient is higher than a certain threshold, profit under centralized scenario is lower than that under decentralized scenario. Due to these two cases, we respectively design revenue sharing contract with franchise fee and wholesale price contract with franchise fee and government subsidy to achieve coordination.

*3. The contribution of this study compared to previous recent studies is quit questionable because some recent publications in this area are missing. Although I know there are some differences between the current work and previous ones, it cannot be guaranteed if the previous literature is not included in your review properly. For example, the developed models should be compared to the following models in the literature review section.*

**Reply:** Thanks for your suggestion. Due to your suggestion, we think this paper is lake of researches related to operational decisions in dual-channel supply chain. Thus, in the revised manuscript, we have added some related references on operational decisions in dual-channel supply chain (p.5), which is also given in the following.

27. Heydari J, Govindan K, Aslani A. Pricing and greening decisions in a three-tier dual channel supply chain. International Journal of Production Economics. 2019; 217: 185-196.

28. Heydari J, Aslani A, Sabbaghnia A. A collaborative scenario-based decision model for a disrupted dual-channel supply chain. Benchmarking: An International Journal. 2019; article in advance, doi: 10.1108/BIJ-06-2019-0281.

*4. It is necessary to show that Hessian matrix (A2) is negative definite by calculating both first and second principal minors.*

**Reply:** Thanks for your comment. In the revised manuscript, we have added the proof that Hessian matrix (A2) is negative definite (p.19), which is also given in the following.

From (A2), we have $\left(-\frac{8 \theta-2 \alpha\theta}{8\theta\left(1-\theta\right)}\right)\cdot\frac{4 \alpha-3 \alpha\theta+4 \theta-8}{4\theta\left(1-\theta\right)}-\left(\frac{8 \theta-2 \alpha\theta}{8\theta\left(1-\theta\right)}\right)^{2}=\frac{\left(2-\alpha\right)\left(4-\alpha\right)}{4\theta\left(1-\theta\right)}>0$ and $-\frac{8 \theta-2 \alpha\theta}{8\theta\left(1-\theta\right)}<0$, which indicates that Hessian matrix of $V_{m}^{D}$ is negative definite.

Thank you again for your constructive comments and valuable suggestions which improve our paper.

**Reviewer 3:**

*The paper titled ‘Coordination contract design in a dual-channel supply chain where the manufacturer has corporate social responsibility?’ seems interesting to me and can be reported through PLOS ONE. However, I suggest that the authors introduce the following major comments to enrich the quality of the manuscript.*

**Reply:** Thanks for your thorough comments and helpful suggestions. Following your suggestion, the issues have been addressed.

*1. The title of the paper seems not okay to me. Modify it.*

**Reply:** Thanks for your suggestion. In the revised manuscript, we have revised our title as “Pricing and coordination in a dual-channel supply chain with a socially responsible manufacturer” (pp.1-2).

*2. Please make sure that a competent editor checks the English. For example, in Line 155 and 229, page 6 and Line 229 respectively. Use of the first person ("I", "we", etc.) and third person ("he", "she" etc.) must be avoided.*

**Reply:** Thanks for your suggestion and we are sorry for the inappropriate expression. In the revised manuscript, we have corrected the inappropriate expression. Besides, we have checked the whole paper to avoid such inappropriate expressions.

*3. The author has used very long sentences due to which it creates a readability problem. Try to make maximum of 11 words.*

**Reply:** Thanks for your suggestion. In the revised manuscript, we have revised long sentences to short ones.

*4. In mathematical expression, small brackets have been used only. In present version, I think this might confuse the readers.*

**Reply:** Thanks for your suggestion. In the revised manuscript, we have revised the expression of the relative equations.

*5. The major defect of the proposed model is that the numerical illustration and sensitivity analysis has not been demonstrated. Also, this study is a lack of managerial implications.*

**Reply:** Thanks for your suggestion. First, we have conducted some numerical examples to illustrate the conclusions intuitively. But we did not put them in a individual section, which is also a general practice in the literature [6, 26]. Second, in the revised manuscript, we have added some managerial implications in the conclusion section (p.16).

*6. I would like to request the author to emphasis on the contributions on practically.*

**Reply:** Thanks for your suggestion. In the revised manuscript, we first have revised the abstract to highlight the contribution of this paper (p.2). Second, we also have revised proposition 2 to emphasis that with the impact of CSR, inconsistency of decision objectives leads to lower profit of supply chain members and lower social welfare, and to analyze the reason caused the above loss (p.11). Third, we also have reorganized the purpose of the socially responsible manufacturer to design contract to highlight the contribution of this paper (p.12).

*7. Line 168, page 7, consumer utility function has been taken. Please cite this equation from which the journal is adopted.*

**Reply:** Thanks for your suggestion. In the revised manuscript, we have cited the following paper for consumer utility function.

37. Chiang W Y K, Chhajed D, Hess J D. Direct marketing, indirect profits: A strategic analysis of dual-channel supply-chain design. Management Science. 2003; 49 (1): 1-20.

*8. The conclusion section is not explained effectively manner. Rewrite it.*

**Reply:** Thanks for your suggestion. In the revised manuscript, we have revised the conclusion section (p.16), which is also given in the following.

We find that the manufacturer’s CSR is a main factor influencing its pricing strategy. When the manufacturer is more socially responsible, it will cut direct price to promote the increase of consumer surplus and product sales. It suggests the manufacturer that taking consumer surplus into consideration and bearing relatively high CSR can be an effective way to expand market share. We also find that the manufacturer’s CSR affects the stability of supply chain structure, which is also the main contribution of this paper. When the manufacturer is less socially responsible, revenue sharing contract with franchise fee can coordinate the DCSC. When the manufacturer has higher socially responsible, the internal transfer contract fails to achieve coordination, which indicates the dual-channel strategy will be resisted by the traditional retailer. However, due to bearing CSR, the manufacturer may receive an external subsidy. For example, the Chinese government rewards enterprises for their social responsibility in developing new energy resources; Gates Foundation offers subsidy for anti-malarial medicines. In this case, wholesale price contract with franchise fee and government subsidy coordinates the DCSC and obtains the largest social welfare.

*9. Literature review is not adequate. The authors can cite the following interesting papers.*

*Cachon, G.P., Lariviere, M.A., 2005. Supply chain coordination with revenue-sharing contracts: strengths and limitations. Manage. Sci. 51, 30–44.*

*Ranjan, Amit, and J. K. Jha. "Pricing and coordination strategies of a dual-channel supply chain considering green quality and sales effort." Journal of cleaner production 218 (2019): 409-424.*

**Reply:** Thanks for your suggestion. In the revised manuscript, we have cited the papers mentioned in your comment in section 2.2 (pp.5-6).

Thank you again for your constructive comments and valuable suggestions which improve our paper.
